# Evaluating the implementation of home delivery of medication by community health workers during the COVID-19 pandemic in Cape Town, South Africa: a convergent mixed methods study

**DOI:** 10.1186/s12913-022-07464-x

**Published:** 2022-01-24

**Authors:** Robert James Mash, Darcelle Schouw, Emmanuelle Daviaud, Donela Besada, Darrin Roman

**Affiliations:** 1 Division of Family Medicine and Primary Care, Stellenbosch University, Cape Town, South Africa; 2 Health Systems Research Unit, SA Medical Research Council, Cape Town, South Africa; 3 Metro District Health Services, Western Cape Government: Health, Cape Town, South Africa

**Keywords:** Primary health care, Primary care, Community health workers, Medication systems, Chronic diseases, Home delivery of medication, South Africa

## Abstract

**Background:**

Primary care services in South Africa have been challenged by increasing numbers of people with communicable and non-communicable chronic diseases. There was a need to develop alternative approaches for stable patients to access medication. With the onset of the coronavirus pandemic there was an urgent need to decongest facilities and protect people from infection. In this crisis the Metro Health Services rapidly implemented home delivery of medication by community health workers. This study aimed to evaluate the implementation of home delivery of medication by community health workers during the coronavirus pandemic in Cape Town, South Africa.

**Methods:**

A convergent mixed methods study evaluated six implementation outcomes: adoption, feasibility, fidelity, coverage, cost, and sustainability of the initiative. Data sources included routinely collected data, a telephonic survey of 138 patients, an analysis of set-up and recurrent costs as well as 17 descriptive exploratory qualitative semi-structured interviews with 68 key informants.

**Results:**

Over a 6-month period 1,054,657 pre-packaged parcels were sent to primary care facilities, 819,649 (77.7%) were delivered and of those 97,297 (11.9%) returned. The additional costs were estimated as 1.3% of a total health budget of R2,2 billion. The initiative was rapidly adopted as it decongested facilities and protected vulnerable patients. Although it was feasible to implement at scale, numerous challenges were encountered, such as incorrect addresses and contact details, transporting parcels, communicating with patients, having a reliable audit trail, and handling out-of-area patients. All role players thought the service should continue and 42.3% of patients reported better adherence to their medication.

**Conclusion:**

Home delivery of medication by community health workers is feasible at scale and affordable. It should continue, but as one of a menu of options for alternative delivery of medication. The following need to be improved: efficiency of the system, the audit trail, adequate support and resources for community health workers, transport of medication, communication with patients, empanelment of patients, governance of the system and training of the community health workers.

## Background

Health services in low- and middle-income countries (LMIC) are challenged by the scarcity of human resources and the growing burden of chronic diseases [1]. In Africa there are colliding epidemics of communicable and non-communicable chronic diseases [1]. For example, South Africa has at least 3,4 million people accessing anti-retroviral treatment [2] and at the same time 1 in 4 people over the age of 45-years has diabetes [3]. The enormous number of people accessing chronic medication threatens to overwhelm primary care services and undermine care for others. At the same time, patients complain of loss of time and income from queueing the whole day at primary care facilities to collect medication.

In order to respond to this dilemma, the health system initiated alternative delivery systems for people with stable chronic conditions [4]. Many provinces introduced Centralised Chronic Medicine Dispensing and Distribution (CCMDD) to automate dispensing and packaging with delivery via alternative pick-up points such as fast lanes at the pharmacy, churches, community halls, or private pharmacies. Adherence clubs that offered both support and pick-up of medication were popular for people living with HIV [5]. More high-tech solutions were also piloted such as e-lockers and ATM-like pharmacy dispensing units [6, 7].

With the onset of the COVID-19 pandemic there was an immediate need to avoid bringing patients with co-morbidities together as they were more at risk of severe disease [8]. There was also a need to decongest primary care facilities to avoid transmission of the virus and to free up capacity to respond to the surge of patients [8]. In this situation, several provinces considered introducing home delivery of medication by community health workers [9, 10]. Internationally, however, there was little to no evidence on the implementation and effects of such an approach. In South Africa, community health worker (CHW) teams had been created as part of re-engineering primary health care across the country prior to the pandemic [11].

.The Metro Health Services (MHS) in Cape Town decided to introduce home delivery of medication, using an existing network of 2500 CHWs [10]. While this was part of the emergency response to COVID-19 the MHS also saw the potential for this to be an ongoing initiative. To guide future policy and decision-making, the MHS commissioned an evaluation of the implementation of home delivery of medication by CHWs. The evaluation was designed to evaluate the adoption, feasibility, fidelity, coverage, cost, and sustainability of the initiative.

## Methods

### Study design

A convergent mixed methods study combined quantitative and qualitative data over the period April-October 2020. The study was pragmatic in the sense that it evaluated the experience of implementing home delivery of medication in the real world context. The methods included:

- Semi-structured descriptive exploratory qualitative interviews
- Analysis of routinely collected data on home delivery.
- A telephonic survey of patients using a short questionnaire.
- Cost analysis

### Setting

The MHS serves approximately 80% of the 4,6 million population in Cape Town who do not have insurance [12]. Since 2017 the MHS committed to a community-orientated primary care approach through a network of 45 primary care facilities. These facilities linked with a network of 2500 CHWs employed by non-profit organisations (NPO). Each team of 10-15 CHWs served a delineated geographic area and was supervised by a professional nurse (PN) [13]. Each CHW was responsible for approximately 250 households.

The MHS was split into four sub-structures. Each sub-structure had a director, a primary health care manager responsible for the facility-based services and a comprehensive health manager responsible for the community-based services as well as a chief pharmacist.

**Fig. 1:**
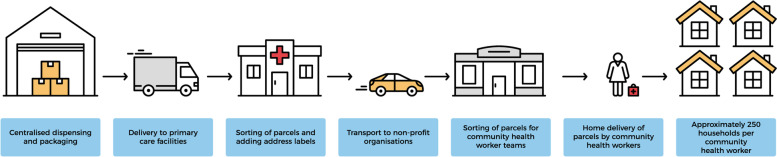
Model of home delivery of medication by CHWs

The model for home delivery of medication by CHWs is shown in Fig. 1. Patients with chronic conditions who were deemed stable were registered with the chronic dispensing unit (CDU). The CDU dispensed and pre-packaged parcels of medication according to the prescription and delivered them to the pharmacy at the primary care facility. Pharmacies had both pharmacists and pharmacy assistants to sort the medication. After sorting at the pharmacy, parcels were transported to the NPO where they were distributed to the CHW teams and delivered to the patients’ homes.

Prior to the pandemic the CHWs were employed to perform household registration and risk assessments, identify and support pregnant women and newborns, promote child and adolescent health, promote family planning, screen for HIV and TB, and support adherence and retention in care for people with chronic conditions. As a result of the pandemic and re-organisation of primary health care, CHWs stopped all routine household registrations and risk assessments and focused instead on screening for COVID-19 and providing information. More time was spent following up clients in the community who would previously have attended primary care facilities. For example, postnatal mothers, people with mental health problems, people with physical care needs, newly diagnosed TB patients, and those needing palliative care. Home delivery of medication was a new activity that was added because of this re-organisation.

### Evaluation of coverage (quantitative data)

Routine weekly data from all facility pharmacists was provided by MHS for August-September (author DR) and included data on the number of parcels sent out by the CDU, number that were sent to CHWs for delivery and number that were returned as undelivered. Data was analysed descriptively as a whole, by sub-structure and per month using the Statistical Package of Social Sciences (SPSS) Version 27.

### Evaluation of costs (quantitative data)

Two health economists (ED and DB) conducted interviews with the sub-structure managers and pharmacists as well as one primary care facility and NPO per substructure. Interviews focused on identifying the set-up and recurrent costs and included a form to detail areas of expenditure. Recurrent costs were based on October when the system had matured and was most representative of future expenditure. Transport costs from facilities to NPOs were modelled on the basis that all transport will be carried out by health services in future. Monthly costs were then annualised. Additional information on expenditure was also sourced from the chief director’s office. Results are presented as additional financial costs and where possible as opportunity costs (e.g. time spent by staff already employed).

### Evaluation of sustainability (quantitative data)

A descriptive telephonic survey of patients who had received home delivery was planned. A sample size calculation (based on error of 5%, confidence intervals of 90%, population of 20,000 and proportion of 75%) gave a sample size of 200, or 50 patients from each sub-structure. The CHWs in the four NPOs that were selected for interviewing (see below), were asked to collect phone numbers from 50 consecutive patients per NPO as they delivered the medication and check that the patient was willing to receive a phone call. Researchers phoned the patients and invited them to answer a short questionnaire in English, Afrikaans, or Xhosa. The questionnaire was designed by RM and validated by the other researchers and chief director’s office. The questionnaire focused on:

- Patients’ overall perception of the value of home delivery
- The advantages and disadvantages of home delivery
- What could be done better
- Preferences for continuing or discontinuing the service

Data was captured in REDCap and exported to SPSS and analysed descriptively.

### Evaluation of adoption, feasibility, fidelity, and sustainability (qualitative data)

A descriptive exploratory qualitative approach using semi-structured individual and focus group interviews was used. Implementation outcomes were explored with key informants across the whole service as shown in Fig. 1. The intention was to interview key informants from MHS management and all four sub-structures. In each sub-structure, we intended to interview informants from one primary care facility and one NPO as well as substructure management. The substructure management selected the primary care facility and NPO. Respondents were interviewed in focus groups or individually, and face-to-face or virtually, depending on what was most convenient. Table 1 shows the key informants that were interviewed.

**Table 1:** Summary of key informants

Focus of interview	Type of interview	Participants	Number of interviews	Number of people
MHS	Individual (virtual)	Medipost manager	1	1
Khayelitsha CHC (SiteB), KESS	Focus group (face to face)	Facility manager, family physician, professional nurse from NPO, CHW based at pharmacy, clinical nurse practitioner. KESS pharmacy manager and Uber coordinators.	1	8
Lotus River CHC, SWSS	Focus group (face to face)	Pharmacist, pharmacy assistant, pharmacist from District Six, facility manager, clinician.	1	5
Delft CHC, NTSS	Focus group (face to face)	Pharmacist, 2 professional nurses, facility manager, CDU champion	1	5
Delft CHC, NTSS	Individual (virtual)	Family physician	1	1
Philani NPO, KESS	Focus group (face to face)	Two CHWs, project manager, dietician, professional nurse.	1	5
Compassionate Action NPO, SWSS	Focus group (face to face)	NPO manager, driver, 2 professional nurses, 2 CHWs. SWSS comprehensive health manager.	1	7
Touching Nations NPO, NTSS	Focus group (face to face)	NPO manager, 5 CHWs, 2 professional nurses.	1	8
Arisen Women NPO, KMPSS	Focus group (face to face)	4 CHWs, 2 professional nurses and NPO manager	1	7
KESS	Individual (virtual)	Primary health care manager	1	1
KESS	Individual (virtual)	Comprehensive health manager	1	1
SWSS	Focus group (face to face)	Primary care manager, pharmacist, medical officer, Uber coordinator	1	4
NTSS	Focus group (virtual)	Pharmacy supervisor, facility manager, comprehensive health manager, primary health care manager, Uber coordinator	1	5
KMPSS	Focus group (face to face)	Pharmacy manager, comprehensive health manager, primary health care manager, Uber coordinator	1	4
MHS	Focus groups (virtual)	Chief director, director of professional support, information management	1	3
MHS	Focus group (virtual)	Two supply chain consultants	1	2
iYezahealth	Individual (virtual)	Manager	1	1
Totals			17	68

*CHC* Community Health Centre, *CHW* Community Health Worker, *MHS* Metro Health Services, *KESS* Khayelitsha-Eastern Substructure, *SWSS* Southern-Western Substructure, *NTSS* Northern-Tygerberg Substructure, *KMPSS* Klipfontein-Mitchells Plain Substructure, *NPO* Non-profit organisation

Interviews were audio recorded in English and a verbatim transcript was checked against the original tapes. Two researchers (RM and DS) independently analysed the data according to the framework method [14] with the assistance of Atlas-ti software.

### Integration of findings

The quantitative and qualitative findings were integrated into one report below based on the implementation outcomes and objectives of the study. Findings were checked by all members of the research team.

### Patient and public involvement in research

Patients and public were not involved in design, collecting data, analysis or interpretation of this research.

## Results

### Adoption

Implementation of home delivery was well aligned with the immediate goals of de-congesting primary care facilities in order to free capacity to handle the surge of COVID-19 patients as well as to reduce the risk of infection amongst people with chronic conditions who would otherwise attend to collect medication:

*“We needed to rapidly decongest our primary healthcare facilities, and the number seemed to indicate that many of the people, I think it was something like 40% of the people, that usually attend our facilities, attended for purposes of collecting chronic medication.” (MHS managers)*

The ability to adopt and implement the initiative quickly, was enabled by the existing CDU and an established network of CHW teams across vulnerable communities. The initiative was also seen as broadly coherent with long term policy goals to strengthen wellness services in communities and become more person-centred.

Such an initiative would usually have been planned and piloted over months, but in this context was implemented in a matter of days. Responsibility for implementation was rapidly decentralised from the chief director’s office, to substructures and to facility and NPO managers. Having such a functional governance structure enabled rapid implementation, although the details of implementation were improvised on the ground in a variety of different ways. Concern was expressed by those responsible for financial and pharmaceutical accountability at the lack of a reliable audit trail to monitor the thousands of parcels. Some senior pharmacists also expressed concern about the legality of distributing medication in this way.

The speed of adoption and implementation meant that there was no time to design electronic systems to support and monitor the process. The system was complex and required coordination between multiple role players to make it work. The widespread availability of WhatsApp became an essential tool in communication and coordination.

### Feasibility and fidelity

Implementation of home delivery and de-escalation of facility-based services for chronic conditions led to a sudden and massive increase in the volume of parcels that needed to be pre-packaged (from 400,000 to 600,000 per month). The CDU had to adapt quickly to demand by working 24/7 and increasing the capacity for manual as well as automated dispensing and packaging. Storage capacity and the size of stock were similarly increased along with the size of vehicles needed to deliver.

Once parcels arrived at facilities, new systems had to be invented to print and attach address labels and sort parcels into geographic areas corresponding to the NPOs. As systems matured the NPOs placed CHWs and nurses at the facilities to sort the parcels into the correct areas. One of the major roadblocks was the lack of reliable patient contact details and addresses.

The health services had not previously needed to ensure this information was up to date. In addition, patients frequently gave false addresses in order to appear within the catchment area of the facility that they wanted to attend. Teams of health workers were identified to phone patients, verify addresses, and ensure consent for home delivery. Unfortunately, most numbers were incorrect:

*“And other than the telephone numbers, the access to outgoing lines was also a challenge. So you could only allocate say you five people in groups. Say group one would draw the folders and then feed group two and then they would do the phoning. But I think there were days when we had like 200 folders and we could only verify 20.” (Lotus River CHC)*

Some facilities decided to only deliver parcels when the address was verified, while others sent out all parcels and followed up those that were undelivered. Addresses were gradually updated as patients attended the facilities, by feedback from CHWs, through the provision of facility-based WhatsApp numbers and through the launch of a metro-wide WhatsApp Bot. Updating the address on the information system at each facility was also a challenge as there were limited computers and people capable of using the software.

Another key constraint was handling out-of-area patients who moved-in with family members during lockdown, had given a false address or attended the facility closest to their work, but not their home. Most facilities found the audit trail too difficult to follow once the parcel was sent elsewhere and could not retrieve the parcel easily. They decided that these patients needed to collect their medication from the facility. It was easier to organise transfer of the parcel when the address was within the same sub-district and some facilities with large numbers of such distant patients persisted with sending the parcels.

Another flaw in the system was due to stock outs when the CDU was unable to supply the medication. Pharmacists at the facility would then attempt to add this medication if they had local stock, although this added more complexity into the organisation of parcels. Communicating the reason for missing medication to patients and what to do was also a challenge, particularly as some patients assumed the CHW had stolen the items.

One missing link in the whole process was how to get medication from the facility to the NPO. Initially this was solved by an arrangement with Uber whereby teams of people at the substructure level coordinated multiple drivers to collect and deliver parcels. While this worked well in the initial crisis the system was unreliable and costly when taken to scale. NPOs complained that parcels arrived too late for them to deliver the same day. Many NPOs eventually used their own drivers to collect the medication:

*“So the most difficult thing with Uber is the drivers to be honest. Once the driver has figured out that they didn’t have an actual passenger in their vehicle, they would take longer routes, they would do other trips, whilst delivering boxes. The most frequent complaint I get from NPOs is that the boxes are being delivered around 9, 10 am when the drivers are actually collecting between 7 and 8 am already.” (SWSS managers)*

Once parcels arrived at the NPO they needed re-sorting to align with the areas served by CHW teams. NPOs would also receive parcels from multiple facilities and needed to integrate these. Initially CHWs would walk to this central point to collect the parcels and return to their areas. This however meant walking long distances, carrying heavy and bulky loads, wasting time which could be better utilised and exposing themselves to possible crime and adverse weather. Most NPOs created secondary drop-off points close to the CHW teams and transported the medication to these points. Some NPOs received and sorted the parcels the day before so that delivery could start immediately the next day.

The PNs who supervised the CHW teams at the NPO were essential to sort parcels at the facility or NPO, use their own cars to transport parcels, oversee the dissemination to CHWs, coordinate delivery and any problems via WhatsApp and cell phone, monitor progress and supervise the return of parcels to the facility as well as collect data on delivery. Some NPOs needed to employ additional CHWs and PNs, with additional funds from MHS, to ensure delivery was possible.

CHWs worked in pairs to improve their security and mostly delivered medication on foot within their designated area. In some cases, drivers assisted for longer distances or more risky areas. The number of parcels gradually increased from 5 to 10 to 20-30 parcels per day, meaning that a large part of the day was spent on this one activity. In some cases, this meant completing deliveries afterhours or on weekends. Safety was a constant concern, although actual incidents were few. A few CHWs were robbed for their cell phone or medication, some were verbally abused or attacked by dogs, or got caught up in community unrest and protest action.

Parcels were returned for several reasons:

- the address was wrong or could not be located,
- the patient was not at home and might be at work,
- the patient had moved to another address,
- the patient had died,
- the medication looked different and the patient did not accept it,
- the patient had a stockpile of existing medication,

There was pressure to return undelivered parcels the same day so that they would be available should the patient come to collect.

Communicating all these changes to patients was problematic and many still came to the facilities to clarify what was happening, causing crowding at the gate. Many facilities introduced WhatsApp lines, which they publicised on the parcels to allow patients to ask questions and get information. This asynchronous communication worked much better than telephone calls, which usually went unanswered by busy reception staff. The metro-wide WhatsApp Bot was also launched to allow any patient to request home delivery or update their address and contact details. While this automated system worked well the subsequent process of verifying and confirming requests was problematic and less effective.

Pharmacies reported daily on the number of parcels delivered and returned and the MHS distributed a weekly dashboard on progress. Parcels did not equate to patients, as patients could receive more than one parcel, thus making it difficult to determine how many people were actually served. Due to the speed of implementation, data from CHWs was all paper based and time consuming to collate and analyse. Some NPOs employed additional data capturers. There was also a dual reporting requirement to the pharmacy at the facility and to the comprehensive health manager at the substructure. Some vertical programmes, such as HIV, also required feedback to determine retention in care.

The audit trail was also paper-based using a variety of bespoke forms, which made it very difficult to determine where the parcel was and who exactly had received it. The lack of an effective audit trail raised practical, legal and financial concerns. Pharmacists who were accountable for the medication felt uncomfortable about this uncertainty. While CHWs were meant to deliver to the patient this was not always possible and medication might be left with relatives.

*“So these are medication in a package, someone’s delivering it, how do you keep control of them, that it doesn't get sold somewhere else, or gets thrown away, or where are some sort of control of the, you know an audit trail, to make sure that you know where the medication is at all times.” (MHS consultants)*

### Coverage

NPOs reported that CHWs were visiting more households as a result of home deliveries than prior to COVID-19. The CDU sent out 1,054,657 parcels over the 6-month period and CHWs attempted to deliver 819,649 (77.7%) of which 97,297 (11.9%) were returned. Overall, they successfully delivered 68.5% of all parcels.

Table 2 shows how delivery varied by month and substructure. The mean percentage of parcels delivered increased from a mean of 70.8 to 80.7%. Likewise, the mean percentage of returned parcels reduced from 11.9 to 8.9%.

**Table 2:** Delivery of PMPs by month and substructure

**By month:**	**Mean number of PMPs delivered per day per SS (SD)**	**Mean % of manifest delivered (SD)**	**Mean % returned (SD)**
April	1068.6 (1058.3)	70.8 (26.2)	11.9 (14.0)
May	1520.8 (803.9)	75.0 (11.4)	22.3 (43.1)
June	1446.7 (655.1)	76.7 (13.0)	18.7 (30.3)
July	1921.4 (805.1)	78.3 (7.9)	11.8 (6.5)
August	1905.1 (907.2)	80.6 (7.9)	9.7 (4.6)
September	1768.8 (886.8)	80.7 (10.2)	8.9 (6.5)
**By substructure:**	**Mean number of PMPs delivered per day (SD)**	**Mean % of manifest delivered (SD)**	**Mean % returned (SD)**
KESS	818.0 (493.1)	84.9 (7.4)	24.0 (36.3)
KMPSS	2270.4 (830.2)	77.5 (8.8)	14.9 (24.7)
NTSS	2114.7 (728.3)	81.0 (8.7)	13.0 (8.9)
SWSS	1294.3 (608.3)	67.7 (17.8)	4.7 (4.1)

*PMP* Patient medication parcel, *SS* Substructure, *KESS* Khayelitsha Eastern Substructure, *SWSS* Southern-Western Substructure, *NTSS* Northern-Tygerberg Substructure, *KMPSS* Klipfontein-Mitchells Plain Substructure, *SD* Standard deviation

### Costs

Set-up costs included creation of the WhatsApp Bot and a communication campaign. The time spent by existing staff at metro, substructure, facility and NPOs in planning home delivery could not be calculated retrospectively.

Additional recurrent costs were incurred through the employment of more CHWs, PNs, drivers and data capturers. Some sub-structures paid overtime to staff. Transport costs were estimated from the costs of Uber and activity of NPO drivers. Community venues were mostly freely available. The WhatsApp Bot had a monthly software maintenance fee. The time spent by existing pharmacy staff (pharmacists and pharmacy assistants) in organising parcels was estimated, although this was not an additional expense.

If the October level of utilisation is extrapolated to the year, annualised set-up and recurrent costs translated into an additional expenditure of GBP1.42 million and opportunity costs of GBP 3.88 million (Table 3). With over 2 million patient deliveries per year (2,027,492), the additional expenditure per patient delivery amounted to GBP0.68 or GBP8.39 per patient year. The value of combined staff time was GBP1.89 per patient delivery. With an annual expenditure for the Metro of GBP106.7 million a year, the additional costs amounted to 1.3% of expenditure (or 5% if opportunity costs included).

**Table 3:** Summary of annual costs (GBP^a^)

	Additional expenditure	Value of existing staff time	Total cost
**Annualised set-up costs**			
Software	4850		4850
Communication campaign	42		42
**Annualised recurrent cost**			
Staff overtime	41,192		41,192
Existing pharmacy staff		318,506	318,506
Additional NPO staff	1290,831		1290.831
Existing NPO staff		3,565,563	3,565,563
Transport	76,348		76,348
Software	8148		8148
**Total**	**1,416,519**	**3,884,069**	**5,300,588**

^a^In this Table 1 GBP = ZAR20.62

### Sustainability

Home delivery enabled decongestion of facilities and undoubtedly prevented infection with COVID-19 in many vulnerable patients. The service therefore most likely saved lives and enabled facilities to have capacity to respond to the surge of people with symptoms of COVID-19. In addition, facility-based professional staff were able to focus more on the needs of unstable patients instead of pushing queues.

As lockdown was lifted, the CHWs were able to perform other activities when they delivered the parcel, therefore increasing the value of the visit. For example, they could perform or update the household assessment (e.g. identify people that had not been retained in care, needed immunisations, had TB symptoms or were pregnant) as well as screen the household for COVID-19. CHWs visited more households in their area and by combining these visits with other activities were able to increase their yield of other health problems. Nevertheless, the increasing time spent on home deliveries as well as working in pairs limited the capacity of CHWs to fulfil their other duties.

Implementation of home delivery strengthened relationships between facility-based and community-based team members and with NPOs. Health professionals as well as community members appreciated the value of the CHWs in offering this service. Closer collaboration with facilities exposed a fault-line in governance as NPOs remained accountable to comprehensive health managers who were not responsible for primary care facilities. Involvement of CHWs in home delivery exposed them to queries from patients about their medication and the need to handle temperature sensitive medication also highlighted the need for additional training.

The researchers phoned 227 patients and 138 (60.7%) answered and completed the questionnaire. The mean age of the patients was 57.7 years (SD 13.3) and 33.3% were male. Almost all patients reported that home delivery was very helpful (111, 81.0%) or helpful (23, 16.8%). Interviewees reported that patients were hugely appreciative of the service:

*“They really appreciate it, and it was, I think for the times, because at the day hospital there is like no order there. Sometimes it’s so, it’s so busy it’s so, then the people wait there, they can get sick there because of what is going on, especially the older people. So to take them their medication it was a joy because we saw how some people was really, really happy and really grateful.” (Arisen women NPO)*

There were teething problems, which generated complaints, and some patients were abusive or uncooperative. Patients also took a while to trust the new system.

Table 4 reports on the advantages and disadvantages of home delivery. The majority reported that home delivery reduced their risk of COVID-19 and saved them money, which could then be spent on food or other necessities. In addition, 42.3% reported better adherence to their medication as a result of home delivery. Many (42.3%) also reported a better experience of the health services as they did not have to spend the day in a queue or expose themselves to crime and bad weather while getting to the facility. Interviewees reported that the visit to the clinic to collect medication was a barrier to many, due to the cost of transport and time spent waiting. For the disabled and elderly it was also a physical challenge to come and collect medication:

*“You know, the patients are getting, they are getting the medication more regularly now. I mean, like, previously, they would have to go there and fetch and they didn't have transport money and didn't have anyone to take them and all that sort of thing, to now they, you know, its ensured that they are getting their medication, because it's being delivered to them.” (NPO)*

**Table 4:** Patients’ feedback on the advantages and disadvantages of home delivery (*N* = 138)

	n	%
**Advantages**		
Reduced my risk of COVID-19	117	85.4
I saved money	73	53.3
Helped me with other issues	55	40.1
I did not run out of medication	64	46.7
I took my medication more often	58	42.3
Not having to go to the facility	58	42.3
Not missing work or school	10	7.3
Improved access for disabled	23	16.8
**Disadvantages**		
I missed my support group	3	2.2
I did not always receive my medication (because parcel was not delivered)	5	3.6
I did not always receive all my medication (because something was out of stock)	26	19.0
The medication looked different to what I was expecting	13	9.5
I had to disclose my illness to other people	0	0.0
I missed seeing people at the clinic	5	3.6
I ran out of medication	14	10.2
I was at a different address	1	0.7
Other	7	5.1
None	83	60.6

In the survey, people with impairments (16.8%), for example with arthritis or being in a wheelchair, found the service particularly valuable. A substantial proportion (40.1%) also reported that the CHW helped them with other issues.

The majority reported that there were no problems with home delivery (60.6%). The main problems were out-of-stock items, items appearing different to what was expected and not receiving the delivery in time.

Everyone interviewed was of the view that home delivery should continue for stable patients on chronic medication. Of the patients surveyed 135 (98.5%) wanted the service to continue in the future and 118 (85.5%) reported they were usually at home and 18 (13.1%) that they were usually at work.

At the same time, most people thought that the future system should be a hybrid with a number of options available to patients. While CHWs should continue with home delivery for some patients, the volume of parcels should be reduced to enable a feasible and comprehensive service.

There were some differences of opinion as to who should receive home delivery in the future. Some thought that the health services should establish criteria, for example, delivery to the elderly, disabled and house-bound:

*“He’s a paraplegic. So for him to come to the facility for parcel collection it’s not possible. He has to pay someone, he has to get, he has to pay someone some ridiculous amount of money to get him here and all of that. So I would focus on the vulnerable patient, the disabled person, the elderly patient.” (Khayelitsha CHC)*

Others thought that patients should select the option that most suited them as, for example, working people might benefit from not losing income, while pensioners might be able to queue at the facility. Some thought that if you establish rigid criteria then you just invite people to innovate ways around the rules. One should not, therefore, decide for people, but rather offer choices. Respondents also felt that these choices should prioritise keeping stable patients out of the primary care facilities.

## Discussion

Home delivery of medication by CHWs was a feasible alternative delivery strategy for stable patients with chronic conditions. It was adopted rapidly in response to the COVID-19 pandemic because of its ability to protect people from infection and to decongest primary care facilities. The initiative can be implemented at scale and is relatively affordable. Both patients and health workers were positive about the initiative and thought it should be continued. Implementation exposed a number of strengths and weaknesses in the primary health care system, which are further discussed below.

Implementation was enabled by a prior commitment to community-orientated primary care [13]. The widespread geographic delineation of vulnerable communities, according to teams of CHWs linked to primary care facilities, enabled rapid implementation. Implementation also strengthened the functional integration of facility-based and community-based members of the PHC team, while exposing a problem with dual accountability in the governance system. The importance of supportive supervision of CHWs by a PN was reinforced and the need for a more functional cell phone based health information system and audit trail.

The initiative was favourably received by health workers and patients and appeared to improve adherence to treatment. Patients spent less time and income on collecting medication and this might have improved household income and food security. These effects, however, have been reported for most of the alternative delivery systems currently in use and are not unique to home delivery [15]. Going forward, home delivery of medication by CHWs should form part of a menu of options for patients.

Such a hybrid system is necessary because home delivery should not replace the other essential activities in the CHWs’ scope of practice – such as health promotion, risk assessment, physical and home-based care [13]. Many of these activities were curtailed during the pandemic, but will resume as soon as possible. A number of options, within the capacity of the health services to manage, will allow patients to select the option best suited to their needs. For example, home delivery may be ideal for frail, elderly, and impaired patients, while alternative pick-up-points as well as e-lockers may be ideal for people that are employed, and adherence clubs for those that need additional empowerment and support with lifestyle change. In some communities, local entrepreneurs were also creating a business out of home delivery for a small fee [16]. All of these options have the advantage of keeping patients out of primary care facilities.

Further efficiencies in the system could be created by delivering parcels direct to the CHW teams rather than via the primary care facilities. The rules for empanelment of patients need to be clarified and systems for updating addresses and contact details improved to enable effective delivery. The accurate registration of patients with primary care facilities is also an essential issue for implementation of national health insurance in South Africa as reimbursement will be based on risk-adjusted capitation [17].

The failure to adequately employ, equip, train, protect and support CHWs has been noted as an issue in implementation of CHW programmes in general [18]. This was also highlighted here with, for example, a need to provide bags, transport, cell phones, and sufficient PNs as well as strategize against crime and violence.

Adequate communication with patients on changes to the organisation of care and the ability to respond timeously to patient queries was exposed by this initiative. In the context of the pandemic, facilities improvised with new technologies such as WhatsApp. Asynchronous communication via cell phone appeared to be a huge leap forward in being responsive to patients in our context. The future automated audit trail should also be integrated with communication to patients on the status of their parcel.

Limitations of the study included the usual problems of routinely collected data. Data collected by CHWs and pharmacists may have mistakes and omissions, however, overall, the data is likely to accurately reflect trends and coverage. It was not possible to interview patients face-to-face during the pandemic and reaching patients via phone was challenging. The sample of patients was biased towards those staying at home and having a successful delivery. Cost data was based on qualitative feedback and recent invoices, but was incomplete, involved numerous assumptions and not all activities could be costed. The large number of qualitative interviews demonstrated similar themes across substructures and saturation of themes was thought to have been achieved. The primary care facilities and NPOs were chosen by the substructures and may have reflected better functioning organisations. If other facilities or NPOs were chosen it is possible that additional themes would have been identified.

## Conclusion

Home delivery of medication by CHWs was adopted in response to the COVID-19 pandemic to decongest primary care facilities and protect patients with chronic conditions. It was rapidly implemented and proved a feasible intervention although numerous challenges were experienced requiring adaptation and innovation. Overall, 69% of all pre-packaged medication across the city was home delivered and only 12% returned. The additional cost was estimated as 1.3% of the total health budget. Patients were positive about the new service and reported improved adherence to medication. All stakeholders agreed that it should continue as part of a range of options for alternative delivery of medication to stable patients with chronic conditions.

## Data Availability

Consent was not obtained to share qualitative interview data outside of the research project. Permission to share routinely collected health service data would need to be obtained from the Western Cape Department of Health. De-identified quantitative data from the patient survey can be shared on reasonable request from the first author, RM.

## Abbreviations

ATM: Automatic Teller Machine
CCMDD: Centralised Chronic Medicine Dispensing and Distribution
CDU: Chronic Dispensing Unit
CHC: Community Health Centre
CHW: Community Health Worker
COVID-19: Coronavirus Infectious Disease 2019
GBP: Pounds
HIV: Human Immunodeficiency Virus
KESS: Khayelitsha Eastern Substructure
LMIC: Low- and middle-income countries
MHS: Metro Health Services
KMPSS: Klipfontein-Mitchells Plain Substructure
NPO: Non-profit organisation
NTSS: Northern-Tygerberg Substructure
PMP: Patient medication parcel
PN: Professional nurse
SWSS: Southern-Western Substructure
TB: Tuberculosis
ZAR: Rand
